# Quantification of Neoagaro-Oligosaccharide Production through Enzymatic Hydrolysis and Its Anti-Oxidant Activities

**DOI:** 10.3390/molecules23061354

**Published:** 2018-06-05

**Authors:** Shu-Ying Xu, Jie Kan, Zhong Hu, Yang Liu, Hong Du, Guang-Chang Pang, Kit-Leong Cheong

**Affiliations:** 1 Guangdong Provincial Key Laboratory of Marine Biotechnology, STU-UNIVPM Joint Algal Research Center, Department of Biology, College of Science, Shantou University, Shantou 515063, China; 17syxu@stu.edu.cn (S.-Y.X.); 13jkan@stu.edu.cn (J.K.); hzh@stu.edu.cn (Z.H.); liuyanglft@stu.edu.cn (Y.L.); hdu@stu.edu.cn (H.D.); 2 Tianjin Key Laboratory of Food Biotechnology, Faculty of Biotechnology and Food Science, Tianjin University of Commerce, Tianjin 300134, China; pgc@tjcu.edu.cn

**Keywords:** *Gelidium amansii* agar, neoagaro-oligosaccharides, β-agarase, anti-oxidant

## Abstract

Neoagaro-oligosaccharides (NAOS) have health benefits that are related to their amount and degree of polymerization (DP). However, the current methods that are used to quantify enzymatically released NAOS are un-specific and time-consuming. Agar has been extracted from *Gelidium amansii* and has been degraded by AgaXa (a recombinant β-agarase). Polysaccharide analysis using carbohydrate gel electrophoresis (PACE) has been adapted in order to quantify NAOS. In addition, the anti-oxidant activity of the degraded samples has been assessed. We have found that the PACE method provided sensitive, precise, and accurate quantification for each of the six NAOS samples. PACE has revealed that the DP of the enzymatic products from the AgaXa digestion were mainly neoagaro-octaose and neoagaro-decaose. The degraded samples exhibited increased radical-scavenging activity towards 2,2-diphenyl-1-picrylhydrazyl and 2,2-azino-bis(3-ethylbenzothiazoline sulfonic acid) radicals. While the anti-oxidant activity may have been from NAOS activity and contributions from neoagaro-octaose and neoagaro-decaose. The adapted PACE method that has been presented here is promising for large sample analysis during quality control and for characterizing novel β-agarase degradation mechanisms.

## 1. Introduction

*Gelidium amansii* is a widely distributed marine algae from the Gracilariaceae (Rhodophyta) family, whose members provide the main source for agar manufacture [1]. In Asia, agar has long been generally recognized as safe and has served as a food additive and gelling agent in pudding, jelly, marshmallow, and other sweets because of its distinct physico-chemical properties. Nevertheless, few studies have examined *Gelidium amansii* for the anti-oxidant potential of these oligosaccharides.

**Figure 1:**
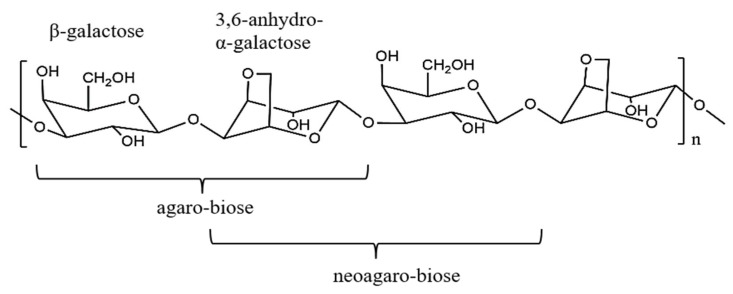
Schematic structure of agaro-oligosaccharides (AOS) and neoagaro-oligosaccharides (NAOS).

Agar is composed of (1–4)-linked 3,6-anhydro-α-l-galactose alternating with (1–3)-linked β-d-galactopyranose, and it can be degraded into two types of oligosaccharides, namely, neoagaro-oligosaccharides (NAOS) and agaro-oligosaccharides (AOS) [2]. However, few NAOS occur naturally, so it is typically generated industrially by hydrolyzing agar using either a chemical or an enzyme. Chemical hydrolysis is economical and simple but it is non-specific and it can produce undesirable by-products, such as toxic furfural and monosaccharides. The enzymatic hydrolysis of agar is generally preferable because it is highly specific, more environmentally friendly, and does not require special equipment [3]. β-agarase is the predominant agarase that can be isolated from marine algae, seawater, and marine microbes [4]. It can cleave the agars’ β-1,4-galactosidic linkages to release NAOS (Figure 1). In contrast, AOS are produced from agar that has been digested with α-agarase [5].

Recently, many reports have characterized the biological roles of NAOS, including its anti-oxidant [6], probiotics [7], anti-obesity [8], and anti-tumor [9,10] activities. Usually, the bioactivity of NAOS correlate closely to their amount and degree of polymerization (DP). Nevertheless, the antioxidant activity of these oligosaccharides don’t have a linear correlation with their DPs generally [11]. It was reported that several antioxidant mechanisms of these oligosaccharides existed, as follows: (a) eliminating the free radicals directly; (b) inhibiting the generation of free radicals; (c) resisting the activation of the oxidation system [12]. Therefore, the popularity of NAOS have greatly increased because of their ability to improve the quality and flavor of many foods. Since NAOS have become increasingly important in the food industry, a robust and reliable method for their quantification in foodstuffs and agar degradation products is needed.

The colorimetric dinitrosalicylic acid (DNS) assay is the traditional analytical method for the quantification of NAOS that has been produced from agar by the action of β-agarase [3,6]. While it is cost-effective, the DNS assay fails to accurately determine the DP of NAOS, because it measures all of the reduced sugars that are present in the solution [13]. A high-performance liquid chromatography (HPLC) and high-performance anion-exchange chromatography-pulsed amperometry detector (HPAEC-PAD) have been used to separate and quantify NAOS [14,15]. Chromatography is a highly precise, but difficult to scale-up, since analyzing multiple samples at one time and requires specific instrumentation [16,17]. These disadvantages have been overcome by polysaccharide analysis using the carbohydrate gel electrophoresis (PACE) method. The reducing end of oligosaccharides are labeled with a fluorophore using reductive amination and are then separated by polyacrylamide gel electrophoresis [18,19]. The attractive features of PACE include parallel sample processing for high sample throughput, robust and simple analysis, and little need for specialized equipment.

The aim of this study was to develop a method to prepare NAOS, with different DP values, from *G. amansii* agar that had been degraded by β-agarase. The different DPs of NAOS were separated by gel electrophoresis and were quantified by fluorescence intensity. Additionally, the anti-oxidant activity of DP on NAOS was investigated. This study details a high-throughput and sensitive method for separating and quantifying NAOS and its DP, which should greatly facilitate the application of NAOS to food products and their quality control.

## 2. Results and Discussion

### 2.1. Sample Pretreatment

To remove the small molecular weight compounds, lipids, and colored matter, the *G. amansii* powder was first treated with an organic solvent. After alcohol precipitation, dialysis, and lyophilization, the dried *G. amansii* agar (7.8 g), free from small molecule sugars, was obtained. The molar mass distribution was between 1.21 × 10^4^ Da and 1.85 × 10^5^ Da. The majority (96%) of the monosaccharide was mainly composed of galactose.

### 2.2. Method Validation of PACE

The PACE method was validated as shown in Table 1. A serial dilution of NAOS, with different DPs, was used to establish the concentration from a standard curve. The equation of the linear calibration curves was calculated by scanning the peak area and converting it into the concentration of each analyte (mg/L). The investigated compounds showed good linearity (R^2^ > 0.9938) within the test range.

The LOD and LOQ values of the four investigated NAOS were between 2.5–3.7 mg/L and 7.9–12.3 mg/L, respectively. The accuracy data for the assay following the determination of each NAOS has been summarized in Table 2. The accuracy was determined by interpolation of the replicate (*n* = 6) peak areas from the accuracy standards of different concentrations and was calculated using a calibration curve. In each case, the relevant error and accuracy percentages were calculated and were less than 6.5% for each NAOS.

The precision was expressed as instrumental, identical, and different gel precision (Table 2). The instrumental precision was assessed by imaging the same gel of the investigated compounds in the standard mix; the overall RSDs were less than 5.5% (*n* = 6). The identical gel precision was determined by analyzing six spots of the mixed standard solution on one gel, while a different gel precision was tested by determining one spot of the mixed standard solution per gel on six gels. The overall RSDs of the identical and different gels were less than 4.3% (*n* = 6) and 4.7% (*n* = 6), respectively. The working solution of the 8-aminonaphthalene-1,3,6-trisulfonic acid (ANTS)-derivatized samples were stored at −20 °C during the analytical procedures and were found to be stable after six days of storage. The samples remained stable in the gel for 30 min after electrophoresis. The recovery of the analytes ranged from 93.7–101.0% (Table 2). Therefore, the PACE method was sensitive enough for the quantitative determination of NAOS, and it was precise, reproducible, and accurate.

It was reported that the validation of the high-performance liquid chromatography (HPLC) showed higher precision with RSDs of under 3.0% [20], which suggested that the HPLC was more precise than the PACE method for quantification. However, the PACE was an effective, sensitive, time-saving and inexpensive method [18], which allowed for the parallel analysis of 15 samples per gel, or the quantification of 14 samples plus one lane of the NAOS mixed solution. The time to complete one run was roughly 30 min. These results demonstrated the great potential of this technique as a result of its ability to quickly and simultaneously assess many different samples on a single gel [18,19]. 

### 2.3. Application for the Quantification of NAOS

**Figure 2:**
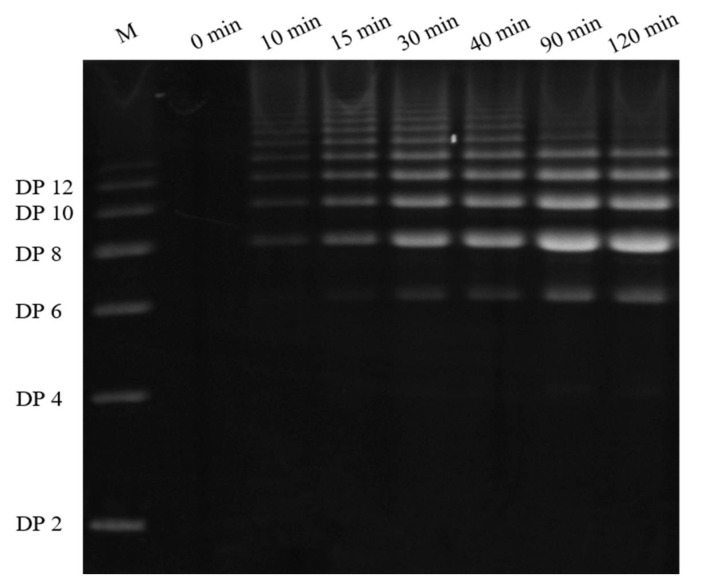
Time course of agar digestion analyzed by polysaccharide analysis using carbohydrate gel electrophoresis (PACE). M indicates the neoagaro-oligosaccharide standards. DP2—neoagaro-biose; DP4—neoagaro-tetraose; DP6—neoagaro-hexaose; DP8—neoagaro-octaose; DP10—neoagaro-decaose; DP12—neoagaro-dodecaose).

The amount of NAOS in each of the digested samples was determined according to the PACE methodology that has been described above. The typical chromatogram and densitograms of PACE are shown in Figure 2. Based on the results that are shown in Table 3, no NAOSs were detected in the agar before AgaXa digestion, and the NAOS concentration increased after digestion. After a long period of digestion, the neoagaro-octaose and neoagaro-decaose were the predominant products, with a concentration of 12.3–13.3 mg/L after 10 min of digestion and 134.9–170.3 mg/L after 120 min of digestion. In addition, the 2 h digested sample only had trace amounts of NAOS with lower DPs, including neoagaro-tetraose and neoagaro-hexaose. No neoagaro-biose was detected after the extended AgaXa digestion time. Recently, various types of β-agarases were isolated from many environmental sources, including seaweed, seawater, marine mollusks, and soils [4]. The β-agarases that were derived from these different sources had different hydrolytic mechanisms, but they primarily produced shorter end products (e.g., neoagaro-biose and neoagaro-tetraose) [21]; in contrast, AgaXa produced longer end products (e.g., neoagaro-octaose and neoagaro-decaose) [22]. 

The DNS assay has been widely utilized and recommended by the International Union of Pure and Applied Chemistry (IUPAC) for estimating the reducing sugar concentration [23]. Therefore, the DNS method was used to determine the concentration of NAOS and to validate the results that were obtained using the PACE method. The total reducing sugar concentrations were found to agree with those from PACE, in some cases (Table 3). However, the DNS assay could not detect the concentrations of the different DPs of NAOS within a digested sample. The PACE method could not only could and discern the concentrations of different DPs of NAOS within one sample, but it could also identify and investigate the enzymatic products. For example, Barton et al., established the enzymatic fingerprinting of pectic polysaccharides from *Arabidopsis thaliana*, using PACE [24]. Overall, the PACE method produced more specific results than the DNS assay in this study.

### 2.4. Anti-Oxidant Activity of NAOS

**Figure 3:**
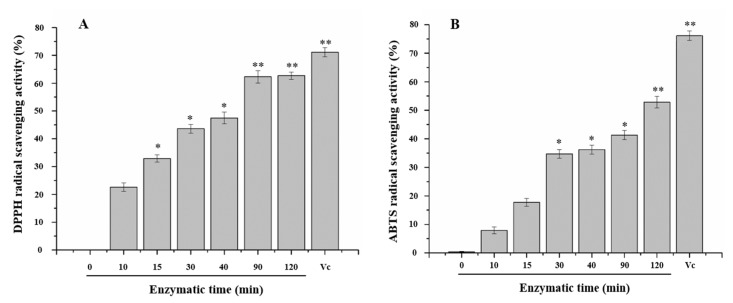
Scavenging ability of 2,2-diphenyl-1-picrylhydrazyl (DPPH) (**A**) and 8-aminonaphthalene-1,3,6-trisulfonic acid (ANTS) (**B**) radicals of the digested sample. A concentration of 200 mg/L of ascorbic acid (Vc) was used as the positive control. * Shows statistically significant differences from value at 0 h, *p* < 0.05; ** Shows extremely significant differences from value at 0 h, *p* < 0.01 (*n* = 3).

In order to establish a relationship between the anti-oxidant activity, NAOS concentration, and DP, the scavenging ability of the 2,2-diphenyl-1-picrylhydrazyl (DPPH) and 8-aminonaphthalene-1,3,6-trisulfonic acid (ANTS) radicals within each digested sample was investigated. Figure 3 shows the inhibitory effect of NAOS that was digested from agar on DPPH and ABTS, compared with 200 mg/L Vc, and suggested that the samples had a greater scavenging radical activity correlated with digestion time. Actually, connected with Table 3, the results indicated that the radical scavenging activity of NAOS was concentration-dependent in all of the concentrations’ tests. Prior to AgaXa digestion, the agar did not exhibit measurable scavenging radical activity. However, after a 10 min digestion, the samples had a total NAOS concentration of 25.6 mg/L and exhibited 20% radical scavenging activity, which suggested that NAOS likely conferred a significant anti-oxidant activity. The activity of NAOS at 200 mg/L (at 30 min incubation time) was lower than that of 200 mg/L Vc. When the total NAOS concentration reached 373.7 mg/L after 90 min of digestion, the free radicals were reduced by over 50%, which further suggested that NAOS may have been positively correlated with anti-oxidant activity. 

The anti-oxidant activity appeared to be related to the types and molecular weights of the carbohydrate that was present in the sample Luo et al. [25]. For example, AoXue Luo et al., isolated and purified four polysaccharides from *Dendrobium nobile*, one of which showed the strongest anti-oxidant activity in the ABTS, DPPH, and Hydroxyl radical scavenging assay, but it possessed the lowest molecule weight [26]. This further demonstrated that the PACE method was superior to the DNS assay because we would not have been able to obtain such results using the DNS assay alone. It also appeared that anti-oxidant activity was related to the presence of the predominant AgaXa enzymatic products, neoagaro-octaose and neoagaro-decaose. This study’s observations on the anti-oxidant activity of NAOS were similar to the findings of Kang et al., who noted that the agaro-oligosaccharides that were degraded by Celluclast exhibited DPPH and ABTS radical scavenging and that this effect was likely dependent on the extent of the agar hydrolysis [6].

## 3. Materials and Methods

### 3.1. Materials and Chemicals

The *G. amansii* was purchased from a seafood market in Shantou, Guangdong, China. The *G. amansii* was cut into small pieces and dried in an oven at 45 °C for 48 h. The dried *G. amansii* was pulverized into a fine powder.

The sodium cyanoborohydride (NaCNBH_3_), 8-aminonaphthalene-1,3,6-trisulfonic acid (ANTS), 2,2-diphenyl-1-picrylhydrazyl (DPPH), and 2,2-azino-bis(3-ethylbenzothiazoline sulfonic acid) (ABTS) were purchased from Aladdin Chemistry Co., Ltd. (Shanghai, China). The other reagents were of an analytical grade.

The recombinant agarase (AgaXa) was obtained from the gene-engineered *Escherichia coli* BL21, carrying the plasmid of pET-32a-AgaXa [27,28].

### 3.2. Preparation of Agar from G. amansii

The *G. amansii* powder (100 g) was extracted twice, with 500 mL methanol/dichloromethane/water (4:2:1; *v/v/v*) aqueous for 12 h at room temperature, with stirring. The residue was collected by filtration and was dried in an oven at 45 °C for 24 h. The dried residue was extracted by 50-fold water at 95 °C for 2 h. Then, the supernatant was concentrated by vacuum and dried at 60 °C until 1 L was formed. Subsequently, 95% ethanol (*v/v*) was added to a final concentration of 70% (*v/v*), and the solution was stayed overnight at 4 °C. The precipitate was collected through centrifugation (4000× *g*, 15 min) and was re-dissolved in hot water and then freeze-dried, which generated *G. amansii* agar.

### 3.3. Enzymatic Digestion of Polysaccharides

The enzymatic hydrolysis of *G. amansii* agar was conducted using the shake flask method. The agar (25 mg) was dissolved in 10 mL 0.02 mol/L Tris-HCl (pH 7.4), which contained agarase (AgaXa). The AgaXa was prepared as a final concentration of 10 U/mL in a 10 mL agar solution. The reaction mixture was incubated at 50 °C for 4 h. Then, the mixtures were heated at 90 °C for 15 min to denature the enzymes. After centrifugation (4000× *g*) at room temperature for 10 min, the supernatants were evaporated to dryness under a stream of nitrogen.

### 3.4. Determination of Reducing Sugar Content

The content of the reducing sugar was determined using the DNS method, with galactose as the standard [29]. The assay was carried out in 96-well PCR plates and was calibrated with galactose standards from 0–0.5 mg/mL. The samples or series standard solution were added to the DNS solution (100 μL) and distilled water (100 μL). The mixture was boiled for 15 min, and after cooling in an ice bath, 0.8 mL of distilled water was added. The absorbance was measured at 540 nm. The content of the reducing sugar in the sample was calculated based on a standard curve of galactose. 

### 3.5. Fluorophore-Assisted Carbohydrate Electrophoresis 

The dried products were derivatized with the 8-aminonaphthalene-1,3,6-trisulfonic acid (ANTS) approach, as described previously by Jackson et al. [30]. In short, 50 μL of 0.1 mol/L ANTS (dissolved in 15% (*v/v*) aqueous acetic acid) and 50 μL of sodium cyanoborohydride (NaCNBH_3_, dissolved in DMSO) were added in reaction mixture, mixed, and reacted under 37 °C for 17 h, then it was dried into dryness using nitrogen gas.

Once ANTS-derivatized, the samples were re-suspended in 0.5 mL of 6 mol/L urea solution. The samples were electrophoresed in a vertical slab gel electrophoresis apparatus, with 10 cm plates, 1.0 mm spacer, and a well with a width of 0.3 cm. The electrophoresis of 32% (*w/v*) polyacrylamide in the resolving gel, with a stacking gel of 8% (*w/v*) polyacrylamide was used for the separation of the enzymatic hydrolysates. The electrophoresis buffer was 0.1 mol/L pH 8.2 of Tris-boric acid. The samples were electrophoresed at the voltage of 300 V for 60 min until the bromophenol blue (migration indicator) had moved to the designed level. The image of the gel was recorded with a charge-coupled device (CCD) camera (Epi-UV FA1100, Aisin Cosmos, Aichi, Japan) under UV 365 nm. The densitometric analysis was carried out using Quantity One software (Bio-Rad Lab, Tokyo, Japan).

### 3.6. Method Validation

For the calibration and assessment of the linearity agaro-oligosaccharides analysis, the mixed agaro-oligosaccharides standard solutions were employed. The linearity was determined by constructing calibration plots (10 μL injected) of the peak area against amounts of each agaro-oligosaccharides (mg/L). The limit of detection (LOD) and limit of quantitation (LOQ) were calculated based on signal-to-noise ratios (*S/N*) of 3:1 and 10:1, respectively. The noise was defined as the peak area that corresponded to the blank solution.

To assess the stability, the peak area was measured by applying each mixed standard solution, electrophoresing the gel, and imaging each picture every 5 min for 30 min. Instrumental precision was checked by imaging the gel of the investigated compound in mixed standard solutions (10 μL) six times. The identical gel precision was determined by analyzing six lanes of the mixed standard solutions (10 μL) on one gel, while different gel precision was tested by determining one lane of the mixed standard solutions (10 μL) on six gels. The accuracy was determined in three different concentrations (low, intermediate, and high) and was calculated as the percent deviation from the nominal concentration.

Recovery (%) was defined as *A_m_/A_s_*× 100%, where *A_m_* was the measured amount, while *A_s_* was the sampling amount. The repeatability was evaluated by six injections of the sample solution in one gel.

### 3.7. Anti-Oxidant Activities of Agaro-Oligosaccharide

The DPPH radical scavenging activity of the samples was measured by the method that was previously reported by Wang et al. [31]. The enzymatic reaction mixture (1 mL, distilled water) was added to 50 mmol/L DPPH in ethanol (1 mL). The mixture was placed in the dark for 30 min, then the absorbance was measured at 517 nm. Ascorbic acid (Vc) was used as a positive control. The DPPH scavenging activity was expressed as the following formula.

DPPH scavenging activity (%) = [1 − (S − Sc)/B] × 100%(1)

where, S, Sc, and B were the absorbance of sample, control, and blank, respectively. The IC_50_ values were defined as the concentration of NAOS that scavenged 50% of the DPPH free radicals.

The ability of the enzymatic reaction mixture that acted as radical scavengers was further tested by an ABTS assay. ABTS radical cation was produced by reacting 7 mmol/L of ABTS in water (ABTS stock solution) with 2.45 mmol/L solution of potassium persulfate, and was stored at room temperature for 12 h. The ABTS solutions were then diluted by a phosphate buffer to an absorbance of 0.70 at wavelength 734 nm. Enzymatic reaction mixtures were added into 4 mL of ABTS. Then, the mixtures were placed in the dark at room temperature for 10 min, the absorbance of the mixtures were recorded at 734 nm. A concentration of 200 mg/L of Vc was used as a positive control. The ABTS scavenging activity was calculated using the same formula as the DPPH radical scavenging activity calculation.

## 4. Conclusions

In this study, a simple method, PACE, was used to detect and quantify NAOS. This study demonstrated that PACE is easy, accurate, and precise and can be used to analyze multiple samples simultaneously. The information that has been obtained from this PACE analysis further elucidates the β-agarase degradation mechanism and also establishes the relationship between NAOS and DPs. The NAOS that had degraded from *G. amansii* agar showed significant anti-oxidant activity and effectively scavenged the free radicals ABTS and DPPH. The Anti-oxidant activity was also influenced by the NAOS concentration and DP. NAOS may serve as a potential nutritional supplement. The data that were generated in this study confirms that the NAOS produced from *G. amansii* agar is an important asset to the food industry.

## Figures and Tables

**Table 1:** Calibration data, limit of quantitation (LOQ), and limit of detection (LOD) of the neoagaro-oligosaccharides.

Analytes	Regression Equation ^2^		*r* ^3^	Test Range (mg/L)	LOQ (mg/L)	LOD (mg/L)
	*a*	*b*				
DP 2 ^1^	1.0563 ± 0.0302	1.3089 ± 0.1128	0.9938	15.0–200.5	12.1	3.6
DP 4	1.7773 ± 0.0428	4.3829 ± 0.3733	0.9955	13.2–185.6	11.8	2.9
DP 6	0.8025 ± 0.0231	0.6730 ± 0.0902	0.9976	12.3–200.4	12.3	3.7
DP 8	1.0226 ± 0.0300	2.0276 ± 0.1536	0.9991	10.1–215.2	7.9	2.7
DP 10	0.4370 ± 0.0137	4.5687 ± 0.4082	0.9945	11.7–210.8	10.0	3.1
DP 12	0.6856 ± 0.0151	1.6525 ± 0.1325	0.9974	15.2–192.7	8.3	2.5

^1^ DP—degree of polymerization; ^2^ Regression equation—Y = aX + b, where, Y is the peak area (Int × mm), X is the concentration of standard NAOS (mg/L), a is the slope, and b is the Y-intercept (*n* = 3); ^3^ *r*—correlation coefficient.

**Table 2:** Precision, accuracy, stability, repeatability, and recovery of neo-agarooligosaccharides.

Analytes	Precision (RSD, %)			Accuracy (%)			Stability	Repeatability	Recovery
	Instrument	Identical Gel	Different Gel	Low	Medium	High	(RSD, %)	(RSD, %)	(%)
DP 2 ^1^	2.9	3.4	3.2	97.3	102.2	98.6	2.3	3.6	99.4
DP 6	0.8	4.3	4.7	98.1	100.6	95.2	2.6	5.3	97.8
DP 8	0.7	3.0	2.4	96.5	99.6	93.8	2.7	3.0	95.7
DP 10	1.2	3.7	3.7	99.5	103.7	95.7	1.2	3.7	97.8
DP 12	2.8	4.3	4.3	93.1	97.0	93.5	3.5	4.3	93.7

^1^ DP—degree of polymerization.

**Table 3:** Concentrations of neoagaro-oligosaccharides in different enzymatic time determined with PACE and DNS.

Method and Analyses	β-Agarase Digestion/Min						
	0	10	15	30	40	90	120
DNS ^1^	4.7	34.8	70.9	188.4	191.6	321.9	355.1
Total NAOS by PACE ^2^ (mg/L)	N.D. ^4^	25.6	101.6	221.8	243.1	373.7	410.2
DP 2 ^3^ (mg/L)	N.D.	N.D.	N.D.	N.D.	N.D.	N.D.	N.D.
DP 4 (mg/L)	N.D.	N.D.	N.D.	N.D.	N.D.	N.D.	N.D.
DP 6 (mg/L)	N.D.	N.D.	Trace	13.3	14.1	36.2	43.6
DP 8 (mg/L)	N.D.	12.3	31.2	70.2	75.2	126.6	134.9
DP 10 (mg/L)	N.D.	13.3	46.1	95.1	109.4	152.5	170.3
DP 12 (mg/L)	N.D.	Trace	24.3	43.2	44.4	58.4	61.4

^1^ DNS—dinitrosalicylic acid; ^2^ PACE—polysaccharide analysis using carbohydrate gel electrophoresi; ^3^ DP—degree of polymerization; ^4^ N.D.—not detected.
